# Reflections on telomere dynamics and ageing-related diseases in humans

**DOI:** 10.1098/rstb.2016.0436

**Published:** 2018-01-15

**Authors:** Abraham Aviv, Jerry W. Shay

**Affiliations:** 1 The Center of Human Development and Aging, Rutgers, The State University of New Jersey, New Jersey Medical School, Newark, NJ 07103, USA; 2 Department of Cell Biology, UT Southwestern Medical Center, Dallas, TX 75390, USA; 3 Center of Excellence in Genomic Medicine Research, King Abdulaziz University, Jeddah, Kingdom of Saudi Arabia

**Keywords:** telomeres, ageing, evolution, cancer, cardiovascular disease, genetics

## Abstract

Epidemiological studies have principally relied on measurements of telomere length (TL) in leucocytes, which reflects TL in other somatic cells. Leucocyte TL (LTL) displays vast variation across individuals—a phenomenon already observed in newborns. It is highly heritable, longer in females than males and in individuals of African ancestry than European ancestry. LTL is also longer in offspring conceived by older men. The traditional view regards LTL as a passive biomarker of human ageing. However, new evidence suggests that a dynamic interplay between selective evolutionary forces and TL might result in trade-offs for specific health outcomes. From a biological perspective, an active role of TL in ageing-related human diseases could occur because short telomeres increase the risk of a category of diseases related to restricted cell proliferation and tissue degeneration, including cardiovascular disease, whereas long telomeres increase the risk of another category of diseases related to increased proliferative growth, including major cancers. To understand the role of telomere biology in ageing-related diseases, it is essential to expand telomere research to newborns and children and seek further insight into the underlying causes of the variation in TL due to ancestry and geographical location.

This article is part of the theme issue ‘Understanding diversity in telomere dynamics’.

## 1. Introduction

The effort to identify persons whose biological age is out of step with their calendrical age has largely fuelled the field of telomere epidemiology. However, only recent research has confronted the deeper and much more relevant question of whether telomere length (TL) might be causal in ageing-related diseases in the general population, which is the focus of this ‘Reflections’ paper. Genetically engineered deficiencies in telomerase and other telomere-maintenance proteins [1–3] have confirmed that short telomeres curtail the lifespan of mice, but only after several generations. That is because inbred strains of mice have very long telomeres; thus, sustained deficiency in telomere maintenance across several generations is necessary to shorten telomeres to a length that impacts health and consequently longevity of these mice.

We can draw a reasonable inference from such mice models to rare human diseases that stem from single detrimental mutations, e.g. dyskeratosis congenita [4] and idiopathic pulmonary fibrosis [5]—genetically inherited diseases marked by critically short TL. These diseases result from germline mutations in telomere regulating proteins, including TERT, TERC, TINF2, WRAP53, PARN, RTEL1, ACD, CTC1, DKC1, NHP2 and NOP10 [6,7]. However, genetically engineered mice have been less successful in modelling the role of telomeres in ageing-related diseases, principally cancer and cardiovascular disease, which are complex human traits driven by multiple genes and gene-environment interactions. Although important, the role of telomere biology in such polygenic traits is typically subtle; it is rarely expressed as a distinct phenotype that in itself explains susceptibility or resistance to a given disease. For these reasons, our present understanding of the connection in the general population between TL and these adult-onset diseases has been largely derived from epidemiology and population genetics, disciplines that generate associative data, which are usually insufficient to establish cause-and-effect relationships (but see §3b).

Here we distil the essence of a large body of research to paint a broad picture of the role of telomeres in ageing-related human diseases. Our focus is on epidemiological research because we believe that insight into the role of TL in human health and disease in the general population has been principally derived from population studies. However, under the section titled ‘The cancer-degenerative-disease trade-off’, we also discuss research in mammals that explains fundamental principles essential for understanding human telomere biology.

TL undergoes progressive shortening in replicating somatic cells *in vitro* and age-dependent shortening in somatic tissues *in vivo*. The popular view regards the pace of this shortening as a ticking biological clock, i.e. a ‘replicometer’. Accordingly, individuals with comparatively short TL are considered biologically older than their peers because their telomere clock is ticking at a faster pace. Based on the premise that ageing and hence short telomeres are deleterious, numerous investigators reported that many detrimental traits and potentially harmful environmental factors are associated with short telomeres in humans. Accordingly, comparatively short TL has become the stand-in parameter for poor health—physical and mental—regardless of the person's age. As we discuss in §3, such a view is short-sighted, since not only short telomeres but also long telomeres might exact a price. Our personal take on the findings that lead to this key conclusion requires understanding the sources of variation in human telomere length, which is covered under a section with this title**.** Finally, we offer the way forward for further research leading to a better understanding of the role of telomeres in ageing-related human diseases.

## 2. Sources of variation in human telomere length

### (a) Variation within somatic cells of the individual and variation across individuals

Since blood is easily accessible, data on the epidemiology and population genetics of TL are principally derived from measurements of TL in blood. Unlike birds, reptiles, fish and other lower vertebrates whose mature erythrocytes are nucleated, human erythrocytes, which outnumber leucocytes by approximately eight hundred to one (available at: http://www.nlm.nih.gov/medlineplus/ency/article/003642.htm, accessed 24 August 2017), have no nuclei. Therefore, only leucocytes are available for TL measurements in the human blood. However, leucocytes comprise multiple lineages and sub-lineages with differing TLs [8,9]. Moreover, somatic tissues display variation in TL that largely arises from their different replicative history, such that minimally proliferative tissues, e.g. skeletal muscle and fat, display longer telomeres than the highly proliferative skin and the haematopoietic system [10,11]. That said, differences in TL across leucocyte lineages [12] and somatic cells [10,11] of the same person are much narrower than TL variation across persons in a given population. It follows that leucocyte TL (LTL), i.e. the mean TL of all leucocytes, represents TL in different leucocyte lineages and somatic cells [10–12]. The upshot is that an individual with a long (or a short) LTL has invariably long (or short) TLs in the majority if not all normal somatic cells. Still, LTL dynamics after birth reflects TL of haematopoietic stem cells and progenitor cells at birth, and telomere shortening due to replication of these cells in the bone marrow and further replication of lymphocytic lineages in the thymus and secondary lymphoid organs [13,14].

### (b) Telomere length dynamics during growth

The rate of TL shortening is considerably faster during early stages of human growth and development than throughout adulthood [8,9]. Most information in this regard is derived from studies in leucocytes. The parsimonious explanation for the rapid LTL shortening during growth is the expansion of the haematopoietic system through replications in tandem with the growing soma [15]. However, with the exception of some information related to skeletal muscle [11], little is known about the pace of TL shortening in somatic cells other than leucocytes. Also minimal information is available about factors other than body mass [11,16] that influence the inter-individual variation in TL dynamics during childhood/adolescence and the extent of their lasting effect on TL in adults.

### (c) The heritability effect on telomere length

The environment (*in utero* and during extra-uterine life) might play a role in fashioning LTL by modifying its pace of age-dependent shortening. For instance, exposure to air pollutants *in utero* [17] and cigarette smoke [18–21] are associated with short LTL. However, a major component of LTL is constitutive. For a given age, approximately 60% of the inter-individual variation in LTL and 30% of its age-dependent attrition are heritable [22–24].

### (d) The sex effect on telomere length

Oestrogen may transcriptionally regulate the catalytic subunit of human telomerase, *hTERT* [25,26]. Therefore, the approximately 150 base pairs longer LTL in women than men [18,20,27–29] has been attributed in part to oestrogen and the presumably slower rate of LTL shortening during the premenopausal period [30]. Although reasonable on the face of it, this notion has been challenged based on longitudinal observations showing that age-dependent LTL attrition is in fact faster in premenopausal than postmenopausal women [31]. Moreover, recent findings indicate that the LTL sex gap is already observed at birth [27] and might be influenced by the gonadal hormonal milieu *in utero* [32].

### (e) The paternal-age-at-conception effect on telomere length

The finding that offspring conceived by older fathers have a longer LTL, i.e. the effect of paternal age at conception (PAC) [24,27,33–35], is an intriguing phenomenon, possibly of fundamental importance for the trans-generational regulation of TL. The PAC effect amounts to approximately 15 base pairs longer LTL per each additional year of the father's age, meaning that an offspring conceived by a 35-year-old man has an average LTL that is longer by 150 base pairs than a similar offspring conceived by a 25-year-old man. This effect on LTL is considerable, given that the average annual rate of LTL shortening in adults is approximately 25 base pairs [36]. The PAC effect might stem from the longer TL in sperm of older men [34,37–39], which is inherited by the offspring in a Mendelian fashion (i.e. in allele-specific manner) [40,41]. The longer TL in sperm of older men has been attributed to high telomerase activity in the testes and presumably the male germ cells in humans and other mammals (telomerase is silent in mature sperm) [42–44]. However, TL elongation in the male germ cells can come about only if telomerase ‘overshoots’ with cell replication, such that TL becomes progressively longer in successive waves of newly formed sperm [39]. Alternatively, ageing might promote selection of male germ cells with a longer TL [35].

### (f) The ancestry effect on telomere length

Sub-Saharan Africans have an average LTL that is longer by approximately 400 base pairs than African Americans [45], whose LTL is longer by about 200 base pairs than that of individuals of European ancestry (Europeans) [29,45,46]. The shorter TL in Europeans might relate in part to polygenetic adaptation due to skin depigmentation that came about with the northbound migration out of equatorial Africa [45]. While pigmented skin renders relative resistance to most forms of cutaneous melanoma, depigmented skin augments the risk of UV light-induced mutations and consequently heightens the risk of melanoma [45,47]. This lethal cancer is more common in Europeans whose LTL is constitutively longer than LTL in controls without the disease [48,49]. Such findings suggest that without polygenic adaptation, which resulted in shorter telomeres in Europeans than sub-Saharan Africans, contemporary Europeans would have been even more susceptible to melanoma than they are at present [45]. A comparatively long TL is probably more advantageous in continental Africa because of a high parasitic/infectious load, which requires a robust immune response throughout the life course. Such a response might draw upon the high proliferative potential due to long TL in cells of the haematopoietic hierarchy.

Moreover, the effect of ancestry on TL may explain in part two puzzling observations. First, while African Americans have more risk factors for cardiovascular disease, they consistently display less atherosclerosis than Europeans [50]. Second, while African Americans have a shorter life expectancy, after the age of 80, their life expectancy is longer than that of Europeans [51,52]. This finding, referred to as the mortality cross-over, is attributed to much lower coronary heart disease mortality in African Americans compared to Europeans [52]. One potential explanation for these observations is the longer TL in African Americans.

## 3. Competing interpretations of the links between telomere length dynamics with ageing and its related diseases

### (a) The conventional view of the ‘clock-like’ nature of leucocyte telomere length *in vivo*

Replication-mediated TL shortening ultimately leads to senescence of human somatic cells in culture [53]; as such, TL reflects the replicative history and replicative potential of cultured somatic cells. These findings have been taken as evidence that in humans TL, as expressed in LTL, serves as a replicometer. Based on this premise, it was proposed that an adult with comparatively short LTL is biologically older than his/her peers [54–56].

This paradigm was further broadened by the concept that the inter-individual variation in age-dependent LTL shortening is largely driven by oxidative stress and inflammation. Studies in cultured cells found that the G triplets of the telomere repeats (TTAGGG) are highly sensitive to the hydroxyl radical [57,58]. Such sensitivity of telomeres to the hydroxyl radical might augment telomere shortening per stem cell/progenitor cell replication, while the inflammatory response might increase replications of haematopoietic stem cells and progenitor cells [54–58]. Together, oxidative stress and inflammation, the hallmarks of adult-onset cardiovascular disease, would therefore augment LTL shortening [57–60]. These putative mechanisms have provided the context for the inference that associations of short LTL with cardiovascular disease [61,62] and early mortality [63–66] in adults are the outcome of a faster age-dependent LTL attrition, principally due to higher burdens of oxidative stress and inflammation. A faster pace of age-dependent LTL shortening has also been invoked in explaining associations of short LTL with a host of human diseases, including psychiatric disorders [67–69].

The evidence supporting the role of oxidative stress and inflammation in the ageing process and the pathogenesis of cardiovascular disease is strong [70–75]. However, given the wide variation in LTL across newborns [27], oxidative stress and inflammation may explain only a small component of the variation in LTL after birth [76,77]. From this perspective, the view of LTL as a clock, whose pace is modified by oxidative stress and inflammation, overlooks the fact that the telomere clock is not uniformly calibrated at ‘zero time’ across newborns and the role of LTL at birth as a principal determinant of LTL throughout the life course [76,77]. It follows that the use of LTL as a marker of biological age might be limited without the ability to scale the individual's LTL to his/her LTL at birth. Moreover, longitudinal studies indicate that adults with more atherosclerotic burden [78] and insulin resistance [79], a risk factor for atherosclerosis, have short LTL but there is no evidence of a higher rate of age-dependent LTL shortening in these individuals compared to peers.

### (b) The alternative view of telomere length as a determinant in ageing-related diseases

Recent research has converged on the premise that TL might be an active determinant in adult-onset disease. The wide TL variation across newborns and children suggests a considerable influence of TL in early life on TL throughout the human life course [11,27]. Such findings do not challenge the potential role of oxidative stress and inflammation during the life course in the association of short LTL with cardiovascular disease. However, they suggest that the overall influence of LTL dynamics during adulthood on LTL may be small compared to LTL at birth and its dynamics prior to adulthood. Consequently, most individuals who enter adult life with short (or long) LTL maintain short (or long) LTL throughout their remaining life [78–80]. In addition, the view that oxidative stress and inflammation explain the association of short LTL with cardiovascular disease has no explanation for findings that long LTL is also associated with some major sporadic cancers [81,82].

Further support for the alternative view comes from genetic analyses. Genome-wide association studies have mapped LTL-associated single-nucleotide polymorphisms (SNPs) to genetic loci, the majority of which harbour LTL maintenance genes [47,83–85]. Genetic risk scores (GRSs) based on these SNPs were developed to predict susceptibility to cardiovascular disease and major cancers. When the GRS predicts short LTL, the probability of developing cardiovascular disease is increased [83,86–88]; when GRS predicts long LTL, the probability of developing major cancers is increased [86,89–93].

Individually, findings of (i) LTL precedence, i.e. having short (or long) LTL is largely determined prior to adulthood, decades before disease onset; (ii) directionality, i.e. short LTL predicts increased cardiovascular disease risk, while long LTL predicts increased cancer risk; and (iii) genetics, i.e. LTL-based GRSs predict disease risk, do not prove causality. However, jointly they provide compelling support for the inference that TL plays a causal role in cardiovascular disease and cancer—the two disease categories that largely define longevity in the US and other middle- and high-income nations.

This conclusion also suggests that mechanisms other than, or in addition to, oxidative stress and inflammation during adulthood might explain LTL variation across the general population and TL disease association. These include diminished replicative potential and perhaps compromised repair ability when TL is short and increased proliferative potential and increased risk of cancer when TL is long [94,95]. Such a paradigm is of particular relevance for cancer, given that telomere biology is at the centre of the interplay between mutations and cell replication dynamics. Moreover, as discussed in the next section, a causal role of TL in cancer and cardiovascular disease aligns with the general notion that evolutionary forces fashion TL in mammals through balancing two diametrically opposing forces: cancer and degenerative diseases.

## 4. The cancer/degenerative-disease trade-off

Evolution is largely driven through interaction between environmental factors and mutagenesis, which is also the key element in the development of cancer. Large mammals (e.g. elephants and whales) have considerably more dividing cells compared with humans and more so compared with mice, which are routinely studied in laboratory settings. Consider a mouse with adult weight of thirty grams and lifespan of 2–3 years versus an elephant with adult weight of six tons and lifespan of 60 years. The ratio of elephant/mouse is 200 000 for body mass and 60 for lifespan. Somatic cells experience, therefore, numerous more replications for growth and maintenance of the elephant's body mass than the mouse's body mass. Since the fidelity of DNA replication is not absolute, everything else being equal, the overall mutational burden, and hence cancer risk, in the elephant would be more than a million-fold higher than that of the mouse. However, Peto pointed out that cancer risk does not always scale with body mass [96]. Evolutionary driven mechanisms that increase cancer resistance in the large mammals—which are typically long-lived—might provide an explanation to the ‘Peto's paradox’ [97].

Recent findings point to increased copy numbers of specific genes that may reduce cancer risk in the elephant and the bowhead whale. In the African and Asian elephants, increased copies of TP53-related sequences (p53) have been detected by genome-wide DNA sequencing [98], and some of these sequences produce functional proteins [99]. Thus, the elephant's resistance to cancer may be in part due to extra copies of a powerful tumour suppressor gene [100]. While the bowhead whale shows no increase in TP53 dosage, its DNA displays increased variants or copies of DNA damage repair genes (mutations in ERCC1 and PCNA and FEN1 duplications) that may account for augmented cancer resistance [101].

Humans are long-lived mid-size mammals. Their somas and those of the elephant and the whale share a dual trait, i.e. repressed telomerase activity and short TL compared to most short-lived small mammals [102]. In principle, short TL/repressed telomerase in somatic tissues would limit replicative potential and attenuate replication-dependent build-up of driver mutations that might bring about malignant transformation. While there are clearly exceptions to this view, the concept of constitutively short TL/repressed telomerase being a cancer protection mechanism has been experimentally tested in a large series of mammals and remains a valid explanation.

Notably, the shortest mouse telomeres are longer than the longest human telomeres. Telomerase knockout mouse cells immortalize with identical frequencies to normal mouse cells and about ten million-fold greater frequency than human cells [103,104]. Apparently, mice and many other short-lived small mammals do not use telomere-based replicative ageing as an anti-cancer protection mechanism. Rather, their long repetitive G-rich telomere sequences might have evolved to serve as a ‘buffer’ that reduces the hydroxyl radical damage to important coding genes [103].

Moreover, genes near telomere ends may be regulated by TL-dependent chromatin interactions, a phenomenon known as telomere position effects over long distances (TPE-OLD). Genes such as human *ISG15* and *TERT* might become dysregulated with progressive telomere shortening. These findings might have implications for how cells turn off telomerase when telomeres reach an optimal length during fetal development and how most cancer cells reactivate telomerase when telomeres become short [105,106]. Thus, replicative senescence due to short telomeres may paradoxically augment cancer risk through telomerase de-repression. We note that such a phenomenon would survive selection because it principally occurs when telomeres are short, i.e. beyond the reproductive period when the force of evolution wanes.

Other studies also indicate that although senescence is an anti-cancer modality, senescent cells secrete a constellation of proteins termed senescence-associated secretory pathway (SASP) proteins, which can lead to dysfunctional tissues and provide a microenvironment that is pro-carcinogenic [107,108]. The clearance of these cells has been suggested to improve tissue function in mice [109,110]. Collectively, these findings indicate that the mere presence of senescent cells might bring about both tissue degeneration and cancer susceptibility through mechanisms that are telomere dependent and independent.

## 5. The way forward

The metaphor that likens telomere dynamics to a ticking clock and the misconception that short telomeres are detrimental while long telomeres are invariably advantageous speaks to the importance of understanding that in the general population telomeres ostensibly converge to an optimal length, which probably strikes a balance between the advantages and disadvantages of having relatively short versus long telomeres. This balance is of particular relevance given that in advanced societies longevity has almost doubled in the last two centuries and in light of an ongoing debate that has left unsettled whether there is a natural limit to the human lifespan [111,112]. Whether or not TL dynamics plays an active role in the ageing process itself, in principle, TL could impose a ceiling on the lifespan of many humans.

It is noteworthy that the overall risks of cardiovascular disease or cancer due to respectively short or long telomeres may be small. However, this might be because the optimal TL strikes a balance between these two disease categories and shifts their impact primarily to the post-reproductive years. All else being equal, an upward or downward drift in the average TL by 1–2 kb would probably result in a respective upsurge in cancer or cardiovascular disease incidence in the general population. For this reason alone, it is of interest to understand the factors that maintain the optimal human TL across generations, the dynamics of which is probably tailored to the genetic makeup in particular environmental settings.

In this context, the overwhelming majority of epidemiological studies have been performed on adults. As the vast variation in TL are observed prior to adulthood, it is important to learn the nature of the factors that influence TL during early development. Moreover, most genome-wide association studies have been performed on Europeans [113]. Thus, extending epidemiological research and genome-wide association studies of TL to non-European populations in their natural habitat and in new settings due to migration will go a long way towards understanding the role of gene–environment interaction in setting the optimal TL and its potential role in ageing-related diseases and longevity.

Finally, the wide inter-individual variation of TL throughout the human life course demands large sample sizes to detect the influence of specific factors (inherent and extrinsic). TL measurements display a high measurement error in the majority of epidemiological studies. While high measurement errors can be offset by increasing sample size, most of these studies have disregarded power considerations indicating that hundreds, if not thousands, of subjects might be needed to obtain reliable LTL results [114,115]. Lack of a systematic attention to LTL measurement error and power limitations explain in part inconsistent findings of association of LTL with a host of traits, including longevity in the elderly, sex and race. It is essential, therefore, that we step back and have a second look at major studies that used flawed design and sub-optimal TL measurements to miss important connections between TL and biological parameters that do exist and detect associations with traits and diseases where none exit.

## Data accessibility

This article has no additional data.
